# Physical activity, screen time and the incidence of neck and shoulder pain in school-aged children

**DOI:** 10.1038/s41598-022-14612-0

**Published:** 2022-06-23

**Authors:** Katariina Pauliina Pirnes, Jouni Kallio, Harto Hakonen, Arto Hautala, Arja Helena Häkkinen, Tuija Tammelin

**Affiliations:** 1 Faculty of Sport and Health Sciences, University of Jyväskylä, P.O. Box 35, 40014 Jyväskylä, Finland; 2 JAMK University of Applied Sciences, LIKES. Piippukatu 2, 40100 Jyväskylä, Finland

**Keywords:** Diseases, Risk factors

## Abstract

This study investigated the associations of accelerometer-measured physical activity, sedentary time and screen time with the incidence of neck and shoulder pain in school-aged children over a two-year follow-up. Children (aged 10–15) were measured at baseline 2013 (T0) (n = 970) and at follow-ups 2014 (T1) and 2015 (T2). Neck and shoulder pain frequency and screen time were determined with a web-based questionnaire. Daytime moderate to vigorous physical activity and sedentary time were measured with an accelerometer. Logistic regression was applied, and the results were adjusted for age, gender, body mass index and bedtime. Accelerometer-measured physical activity or sedentary time at baseline were not associated with the incidence of neck and shoulder pain at the two-year follow-up. Associations of neck and shoulder pain incidence with overall screen time (*p* = 0.020), and especially with passive gaming time (*p* = 0.036) and social media time (*p* = 0.023) were found at the first but not the second follow-up. The neck and shoulder pain incidence associated with overall screen time, passive gaming time and social media time at the first follow-up. The importance of limiting screen time, should be explored in order to find new approaches in preventing neck and shoulder pain in school-aged children.

## Introduction

Research on the predictors of neck and shoulder pain (NSP) in children is lacking despite the fact, that NSP has become one of the most persistent musculoskeletal pain symptoms among school-aged children^1,2^. The increase in NSP among youth was noticed in the Finnish study 1990s, at around the same time as the use of information and communication technology was becoming increasingly common^3^. A similar increase in NSP has also been reported in other western countries^4^. Neck pain is also a common global problem today. The years lived with disability in age standardized rates are the highest in Western Europe and East Asia (461/100 000) in general population according the latest Global burden of disease study 2017^5^.

Identifying the factors influencing the development of NSP in young populations is an important first step in the early prevention of this major public health problem.

There is conflicting information about the long-term factors influencing the NSP. The factors that have been observed to predict the incidence of NSP in children include physical and / or psychological stress, personal hobbies, and the co-occurrence of other musculoskeletal symptoms^6^. NSP has also been found to be hereditary (68%)^6^ and it has been reported that children from families with a history of musculoskeletal pain are 58% more likely than other children to suffer from pain symptoms^7^. High levels of PA have been reported to be associated with a higher prevalence of NSP in girls^8^ but a lower prevalence in boys^9^. In a retrospective study, maintaining PA from childhood to adolescence inhibited NSP^10^. In general, the presence of pain has been associated with reduced PA in school-age^11^. Some studies have found no longitudinal association between spinal pain or NSP and the PA level^6,12,13^. For example, a 25-year follow-up study of originally 9- to 17-year-olds showed good flexibility in boys and endurance strength in girls to be associated with less tension neck symptoms in adulthood but no association between adolescent PA assessed by questionnaire and adulthood tension neck was found^13^. A four-year follow-up of 9- to 12-year-olds found no association between PA level measured by questionnaire and future persistent neck pain^6^. A later study in 11- to 15-year-olds in which PA was measured with an accelerometer also found no association of neck pain with PA in children at the two-year follow-up^12^.

The WHO has identified important areas for further research, one of which is the differences in the health effects of PA and its different types and domains, including ST and screen time^14^. Frequent computer use has been found to be associated with a higher prevalence of NSP in children^8,15^. However, computer-related activities or screen time have not been related to NSP in all studies^16,17^. According to a recent report, problematic use of social media was associated with NSP in children^18^.

Given the mixed evidence for the impact of reduced PA on NSP, this study investigated the longitudinal associations between accelerometer-measured PA and the incidence of NSP in 10–15 -year-old children. The article presents the results of a two-year follow-up, complementing our previous cross-sectional analysis of the same population^9^. The specific aim was to ascertain whether accelerometer-measured PA or ST and self-reported screen time explain the incidence of NSP in school-aged children.

## Material and methods

As a part of a research related to the national “Finnish Schools on the Move” program^9,19–22^, 1710 school-aged children in grades 4–7 across Finland were invited to participate in the study. Of these children, 970 participated (mean 12.5 years ± 1.3 years; 52.5% girls) and 684 (75.6%) provided information on all the study variables at baseline. We utilized data from three measurement points: spring 2013 (T0, baseline), spring 2014 (T1) and spring 2015 (T2).

The incidence of NSP refers to new cases, where pain is reported as having occurred at least once a week during the past three months. Analyzes were conducted on the data obtained between baseline and the second measurement point (T0–T1) and on the data obtained between the second and third measurement points (T1–T2). In accordance with the Declaration of Helsinki, a written informed consent was obtained from all the children and their guardians before participation. The study setting was approved by the ethics committee of the University of Jyväskylä (January 2012).

The participants completed a web-based questionnaire five times during school hours in 2013–2015^21^. The pupils answered the question: “How often have you had the following symptoms in the last three months?” The accompanying list of symptoms included “neck or shoulder pain or ache”. Participants responded to each symptom by selecting one of five pain frequency options: (1) almost daily, (2) more than once a week, (3) about once a week, (4) once a month, and (5) rarely or never. The questionnaire included a figure of the human body divided into named zones to ensure that the body regions were understood correctly. It was possible for all participants to ask for help with the questionnaire from an adult in the classroom. For the incidence analysis, the answers on NSP symptoms during last three months were recoded into two categories: (1) once a week or more often and (2) less than once a week. Children were also asked to report if the pain originated due to a trauma: “Have you injured any of the above-mentioned and pictured pain areas during the previous three months (for example, fallen, stumbled, breached during sport, etc.)?”^21^. Children who reported trauma during last three months in the neck and shoulder area were excluded from the analysis. The test–retest repeatability of the NSP questionnaire has been reported to be substantial (Kappa [κ] 0.68 for the 2-point scale and intraclass correlation coefficient [ICC] 0.67 for the 5-point scale)^21^.

Screen time was asked with five questions, separately for weekdays and weekend days^20^. Children were asked to state how many hours a day they usually spend (1) watching TV, videos (including YouTube and other sources) or DVD movies, (2) playing computer or console games (excluding sports games like Wii Fit, Xbox KINECT or PlayStation Moves), (3) doing homework on a computer or other electronic device (iPad, etc.), (4) communicating with others through social media such as email, SMS, Twitter, Facebook, chat and (5) reading printed or electronic books, magazines, newspapers, etc. outside of class (e.g. at home, during school breaks, at meal breaks etc.). All items were answered on a nine-point scale: (1) not at all, (2) about half an hour a day, (3) about an hour a day, (4) about 2 h a day, (5) about 3 h a day, (6) about 4 h a day, (7) about 5 h a day, (8) about 6 h a day, and (9) about 7 h a day or more. The response options were coded according to the number of hours: 0, 0.5, 1, 2, 3, 4, 5, 6, 7. The screen time items analyzed were TV viewing time, game-playing time and social media time. Test–retest agreement for self-reported screen time questions has been moderate to substantial (ICC 0.54–0.74)^23^.

Daytime PA and ST were assessed 5 times with a hip-worn ActiGraph triaxial GT3X + and wGT3X + accelerometers (ActiGraph, Pensacola, Florida, USA) in 2013–2015^20^. Participants were instructed to wear the monitor on the right hip during waking hours for seven full consecutive days, except while bathing or doing water-based activities. The 30 Hz raw acceleration data were downloaded, standardly filtered and converted to 15-s epochs using Actilife software (version 6.11.7). A customized Visual Basic macro for Excel software was used for data reduction. Readings including ≥ 500 min/day on three days (two valid weekdays and one weekend day) or more of time measured between 7:00 and 23:00 were required for a valid monitoring period. Periods of 30 min of consecutive zero counts were defined as non-wear time. Counts over 20 000 per minute (cpm) were omitted as spurious accelerations^24^. The cut-points proposed by Evenson et al. (2008) were used to calculate MVPA (≥ 2296 cpm) and ST10min (≤ 100 cpm)^25^. Accelerometer-based ST (ST10min) was the absolute wear-time in which at least 10 min were sedentary (< 100 cpm). MVPA and ST10min were calculated as weighted means of the weekday and weekend day means (Total PA = [5 * mean weekday PA + 2 * mean weekend day PA]/7).

Each pupil’s weight and height were measured and used to calculate their body mass index (BMI). Weight was measured in light clothing using bioelectrical impedance analysis (InBody 720, Biospace Co., Ltd). Height was measured with a portable Charder HM 200P instrument. The measurement was performed twice. If the results between the measurements differed by more than 0.4 cm, a third measurement was performed. The mean of the two closest results was used in the analysis. Bedtime was asked with the question: “When do you usually go to bed if you have to go to school next morning”? The response options were selected from a list of times specified in half hour intervals (from 1 to 7): no later than 21:00, 21:30, 22:00, 22:30, 23:00, 23:30 or 24:00 or later.

### Statistical analysis

The variables observed at the three measurement points, i.e., baseline (T0), one-year follow-up (T1) and two-year follow-up (T2) were analyzed. In the analyses MVPA and ST (ST10min) was used as absolute values min/day. Descriptive statistics are presented as mean values and standard deviations (SD) or percentages (%). NSP incidence was calculated as follows: for pupils with NSP less than once week at T0 and T1 the NSP incidence variable assigned the value 0 (reference category); if the participant’s NSP was higher than the reference category value at T1, the NSP incidence variable was assigned the value 1. Children with NSP at least once a week at T0 were not included in the analysis. The unequal probabilities of selection (by age and gender) were controlled for the modeling by using sampling weights, which were constructed by using information on general population structure obtained from Official Statistics of Finland^26^. Model parameters were estimated by using full information maximum likelihood method (FIML)^27^ with robust standard errors (MLR). Missing data were assumed to be missing at random (MAR). Because data were clustered in schools and ages, standard errors were calculated by using a special feature of Mplus (TYPE = COMPLEX). To study the incidence of NSP in the different independent variables adjusted by spinal area injuries (upper or lower back, n = 81), BMI, bedtime (23.00 or later), age and gender, logistic regression analysis with was conducted. Dropout was studied by using the FIML, where the model was corrected with a missing value. The analyzes were conducted using SPSS 25.0 for Windows (SPSS Inc., Chicago, IL) and Mplus 7.0 using a 5% significance level, i.e., p-values ≤ 0.05 indicating a significant association.

## Results

No important differences were found between the participants with (n = 684) or without (n = 286) complete data in all relevant variables. At baseline, 947 children and at the last measurement point 798 children answered the survey. At baseline (T0), 26% of children reported experiencing NSP at least once a week. The prevalence of NSP in girls and boys was 28% and 23%, respectively, at T0 (*p* = 0.091) and 31% and 21% at T2 (*p* = 0.001). The incidence of NSP between T0–T1 was 15% and between T1–T2 18%. The incidence of NSP between T0–T1 was higher in girls than boys (girls 19% vs. boys 11%, *p* = 0.006) but no difference was observed between T1–T2 (girls 20% vs. boys 16%, *p* = 0.207) (Table 1). At T2, mean MVPA among all children was 7.5 min less and mean sedentary time was approximately 1 h greater than at baseline (T0) (Table 1).

**Table 1:** Study variables for all participants and for boys and girls separately at T0, (2013), T1 (2014) and T2 (2015).

	Year	ALL		BOYS		GIRLS		*p* (boys/girls)
		Mean (sd)	N	Mean (sd)	N	Mean (sd)	N	
Age (years)	T0	12.5 (1.3)	970	12.6 (1.3)	462	12.5 (1.3)	507	0.605
BMI (kg/m2)	T0	18.9 (3.2)	914	18.6 (3.3)	429	19.1 (3.2)	485	0.054
Bedtime (23.00 or later)	T0	54 (5.7)	947	24 (5.3)	451	30 (6.0)	496	0.630
Injury at spinal area (%)	T0	81 (8.6)	947	45 (10.0)	451	36(7.3)	496	0.135
NECK AND SHOULDER PAIN NSP total, range 1–5*	T0	2.0 (1.1)	905	1.9 (1.0)	430	2.0 (1.1)	475	0.110
	T1	1.8 (1.0)	847	1.7 (0.9)	400	2.0 (1.1)	447	< 0.001
	T2	1.9 (1.1)	780	1.8 (1.0)	369	2.1 (1.1)	411	< 0.001
NSP (at least once/week) (%)	T0	235(26)	905	99(23)	430	133(28)	475	0.091
	T1	195(23)	847	68(17)	400	125(28)	447	< 0.001
	T2	211(27)	780	78(21)	369	127(31)	411	0.001
NSP incidence %	T0–T1	91(15)	605	32(11)	294	59(19)	311	0.006
	T1–T2	105(18)	586	47(16)	295	58(20)	291	0.207
ACCELEROMETER- MEASUREMENTS MVPA (min/day)	T0	52.7 (21.7)	767	59.2 (23.7)	342	47.5 (18.4)	425	< 0.001
	T1	51.5 (22.6)	503	57.8 (25.0)	209	47.1 (19.5)	294	< 0.001
	T2	45.2 (19.1)	319	47.1 (19.8)	127	43.9 (18.5)	192	0.145
ST10min (h/day)	T0	3.7 (1.2)	767	3.5 (1.3)	342	3.8 (1.2)	425	0.003
	T1	4.1 (1.3)	503	3.9 (1.3)	209	4.3 (1.3)	294	0.007
	T2	4.8 (1.4)	319	4.8 (1.4)	127	4.8 (1.3)	192	0.817
SELF-REPORTED SCREEN TIME Screen time (h/day)	T0	4.2 (2.8)	948	4.5 (3.1)	451	4.0 (2.5)	497	0.002
	T1	4.7 (3.0)	868	5.2 (3.2)	411	4.3 (2.7)	457	< 0.001
	T2	5.0 (3.1)	798	5.4 (3.1)	381	4.6 (3.1)	417	< 0.001
TV time (h/day)	T0	1.8 (1.2)	948	1.9 (1.2)	451	1.7 (1.1)	497	0.088
	T1	1.9 (1.3)	868	2.1 (1.3)	411	1.8 (1.2)	457	0.002
	T2	2.0 (1.3)	798	2.1 (1.3)	381	1.9 (1.2)	417	0.020
Games (h/day)	T0	1.3 (1.3)	948	1.8 (1.4)	451	1.0 (1.0)	497	< 0.001
	T1	1.2 (1.3)	868	1.8 (1.5)	411	0.7 (0.9)	457	< 0.001
	T2	1.2 (1.5)	798	2.0 (1.6)	381	0.5 (1.0)	417	< 0.001
Social media (h/day)	T0	1.1 (1.2)	948	0.9 (1.2)	451	1.3 (1.3)	497	< 0.001
	T1	1.6 (1.6)	868	1.3 (1.5)	411	1.8 (1.7)	457	< 0.001
	T2	1.8 (1.7)	798	1.3 (1.5)	381	2.3 (1.8)	417	< 0.001

BMI = Body mass index; *response options 1 = rare or never, 2 = about once a month, 3 = once a week, 4 = more than once a week, 5 = almost daily; ST = Sedentary time, MVPA = Moderate to vigorous physical activity; *p* value for gender difference (Student’s t-test or Pearson’s chi-squared test).

Self-reported screen time was analyzed as whole and separately for TV-viewing, passive gaming and social media time. During the follow-up, boys accumulated 0.9 h/day of screen time, while girls accumulated 1 h/day of social media time. Mean screen time differed significantly by gender during the follow-up (T0: girls 4.0 h/day vs. boys 4.5 h/day, *p* = 0.002, T2: girls 4.6 h/day vs. boys 5.4 h /day, *p* < 0.001) (Table 1). In the whole sample, MVPA correlated with NSP at baseline and sedentary time correlated with NSP at baseline and T1. A weak correlation between sedentary time and the incidence of NSP between T0–T1 was detected. Screen time correlated with NSP in all children but not with the incidence of NSP. Social media time correlated with NSP at each measurement point (Table 2).

**Table 2:** Pearson correlation coefficients between variables and neck and shoulder pain (NSP) at T0, T1 and T2 for all participants.

ALL	NSP			NSP prevalence (at least once a week)			incidence	
	T0	T1	T2	T0	T1	T2	T0–T1	T1–T2
**MVPA**								
T0	− 0.056	− 0.109**	− 0.108**	− 0.078*	− 0.090*	− 0.099*	− 0.072	− 0.037
T1	− 0.011	− 0.058	− 0.086	− 0.038	− 0.049	− 0.080	− 0.079	− 0.055
T2	− 0.017	− 0.047	− 0.012	− 0.034	− 0.028	− 0.071	− 0.019	0.002
**ST10min**								
T0	0.061	0.126**	0.086*	0.061	0.109**	0.048	0.093*	− 0.008
T1	0.038	0.127**	0.163**	0.040	0.102*	0.147**	0.118*	0.105
T2	− 0.005	− 0.036	0.028	0.011	− 0.023	0.080	− 0.008	0.106
**Screen time**								
T0	0.129**	0.078*	0.101**	0.117**	0.070*	0.104**	0.052	0.052
T1	0.079*	0.116**	0.092*	0.074*	0.097**	0.099**	0.073	0.047
T2	0.088*	0.090*	0.114**	0.126**	0.070	0.101**	0.055	0.098*
**TV time**								
T0	0.031	0.019	0.048	0.035	0.021	0.059	0.001	− 0.016
T1	0.000	0.030	0.031	0.007	0.024	0.031	0.004	0.001
T2	0.031	0.053	0.071*	0.074*	0.056	0.078*	0.007	0.059
**Games**								
T0	0.112**	0.011	− 0.012	0.101**	0.018	0.002	0.010	0.005
T1	0.028	− 0.009	− 0.067	0.016	0.013	− 0.042	− 0.019	− 0.039
T2	0.024	− 0.046	− 0.041	0.042	− 0.033	− 0.042	− 0.014	0.011
**Social media**								
T0	0.146**	0.148**	0.199**	0.126**	0.120**	0.182**	0.109**	0.132**
T1	0.119**	0.194**	0.196**	0.116**	0.147**	0.188**	0.149**	0.120**
T2	0.114**	0.165**	0.190**	0.136**	0.114**	0.162**	0.107*	0.128**

* *p *< 0.04, ** *p* < 0.05, MVPA = Moderate to vigorous physical activity (≥ 2296 cpm); ST10min = sedentary time of at least 10 min (< 100 cpm).

At baseline, 767 children participated in PA monitoring with accelerometers, of whom 319 participated in the second follow-up (T2). Accelerometer-measured MVPA or ST were not associated with the incidence of NSP between T0–T1 or between T1–T2 (Table 3). Self-reported screen time was significantly associated with the incidence of NSP (*b* = 1.88, *se* = 0.81, *p* = 0.020) between T0–T1, but not between T1–T2 (Table 3).

**Table 3:** Results of logistic regression analyses on incidence of neck and shoulder pain (NSP) in relation to independent variables in models. Variables at measurement point T0 or T1 predicting NSP incidence at next follow-up period (T0–T1 or T1–T2).

	NSP incidence T0–T1		95%CI	*p* value	NSP incidence T1–T2		95%CI	*p* value
	b (se)	OR			b (se)	OR		
**MVPA**								
MVPA	− 1.05 (0.81)	0.91	0.79–1.05	0.195	0.98 (0.92)	1.08	0.94–1.25	0.283
MVPA × gender	0.01 (0.22)	1.00	0.97–1.03	0.947	− 0.08 (0.16)	1.00	0.98–1.01	0.604
MVPA × age	1.01 (0.75)	1.01	1.00–1.02	0.174	− 0.90 (0.86)	0.99	0.98–1.01	0.295
Bedtime (23.00 or later)	0.13 (0.05)	4.39	1.75–11.04	0.005	0.09 (0.05)	1.89	0.88–4.08	0.102
Gender*	0.13 (0.24)	1.66	0.29–9.54	0.569	0.19 (0.18)	2.02	0.53–7.74	0.300
BMI	0.00 (0.07)	1.00	0.92–1.08	0.968	− 0.06 (0.07)	0.96	0.89–1.04	0.344
Age	− 0.12 (0.21)	0.83	0.44–1.57	0.564	0.33 (0.21)	1.67	0.89–3.11	0.106
Injury	0.01 (0.04)	1.14	0.47–2.75	0.772	0.13 (0.05)	3.38	1.35–8.44	0.008
**ST10min**								
ST10min	1.02 (0.77)	5.11	0.43–60.20	0.187	− 0.26 (0.84)	0.68	0.05–8.50	0.762
ST10min × gender	− 0.07 (0.26)	0.94	0.57–1.54	0.791	− 0.25 (0.29)	0.81	0.50–1.31	0.392
ST10min × age	− 1.00 (0.81)	0.89	0.74–1.07	0.219	0.36 (1.01)	1.04	0.85–1.28	0.721
Bedtime (23.00 or later)	0.12 (0.05)	3.73	1.53–9.08	0.009	0.07 (0.05)	1.69	0.84–3.42	0.140
Gender*	0.22 (0.26)	2.28	0.31–16.60	0.412	0.33 (0.27)	3.40	0.45–25.51	0.228
BMI	0.01 (0.07)	1.00	0.93–1.09	0.928	− 0.09 (0.07)	0.95	0.87–1.03	0.232
Age	0.34 (0.21)	1.72	0.88–3.36	0.106	0.06 (0.29)	1.09	0.46–2.59	0.841
Injury	0.02 (0.04)	1.21	0.49–3.01	0.678	0.12 (0.05)	2.94	1.19–7.30	0.018
**Screen Time**								
Screen Time	1.88 (0.81)	3.79	1.37–10.47	**0.020**	− 0.75 (0.69)	0.61	0.26–1.47	0.272
Screen Time × gender	− 0.07 (0.10)	0.94	0.79–1.12	0.502	0.06 (0.11)	1.04	0.90–1.20	0.605
Screen Time × age	− 1.85 (0.83)	0.90	0.83–0.98	0.027	0.76 (0.66)	1.04	0.97–1.11	0.254
Bedtime (23.00 or later)	0.09 (0.04)	2.80	1.27–6.20	0.018	0.08 (0.05)	1.74	0.86–3.53	0.124
Gender*	0.24 (0.12)	2.51	0.98–6.43	0.050	0.06 (0.12)	1.26	0.51–3.10	0.614
BMI	0.00 (0.06)	1.00	0.93–1.08	0.945	− 0.07 (0.07)	0.96	0.88–1.04	0.303
Age	0.36 (0.11)	1.78	1.26–2.53	0.001	0.03 (0.11)	1.04	0.75–1.46	0.809
Injury	0.02 (0.04)	1.33	0.54–3.26	0.539	0.12 (0.05)	2.95	1.21–7.18	0.016
**TV**								
TV	0.87 (0.58)	4.45	0.68–29.04	0.132	− 0.86 (0.72)	0.28	0.03–2.32	0.233
TV × gender	− 0.09 (0.11)	0.85	0.57–1.27	0.424	0.09 (0.12)	1.14	0.81–1.61	0.451
TV × age	− 0.84 (0.59)	0.89	0.77–1.04	0.157	0.77 (0.69)	1.09	0.93–1.28	0.264
Bedtime (23.00 or later)	0.11 (0.04)	3.34	1.44–7.73	0.010	0.08 (0.05)	1.81	0.87–3.78	0.110
Gender*	0.23 (0.12)	2.41	0.95–6.12	0.059	0.04 (0.12)	1.16	0.47–2.81	0.750
BMI	0.01 (0.07)	1.00	0.93–1.09	0.925	− 0.07 (0.07)	0.96	0.88–1.04	0.349
Age	0.26 (0.10)	1.51	1.08–2.09	0.013	0.03 (0.12)	1.04	0.73–1.49	0.812
Injury	0.01 (0.04)	1.19	0.50–2.82	0.698	0.13 (0.05)	3.15	1.28–7.72	0.012
**Games**								
Games	2.75 (1.31)	5.79	1.08–30.99	**0.036**	− 0.68 (0.56)	0.37	0.08–1.84	0.226
Games × gender	− 0.18 (0.22)	0.94	0.79–1.10	0.419	− 0.15 (0.08)	0.67	0.45–1.00	0.050
Games × age	− 0.20 (0.10)	0.19	0.04–1.00	0.046	0.69 (0.53)	1.08	0.96–1.22	0.191
Bedtime (23.00 or later)	1.06 (0.39)	1.10	1.02–1.18	0.007	0.08 (0.05)	1.76	0.88–3.51	0.110
Gender*	0.92 (0.40)	1.27	1.04–1.55	0.021	0.18 (0.10)	1.94	0.90–4.16	0.085
BMI	0.00 (0.04)	1.01	0.89–1.13	0.930	− 0.08 (0.07)	0.95	0.88–1.03	0.249
Age	0.44 (0.16)	1.31	1.09–1.59	0.006	0.07 (0.07)	1.12	0.91–1.38	0.291
Injury	0.22 (0.45)	1.02	0.95–1.09	0.627	0.12 (0.05)	2.94	1.17–7.39	0.020
**Social media**								
Social media	1.71 (0.75)	17.71	1.62–193.1	**0.023**	0.34 (0.70)	1.53	0.27–8.57	0.630
Social media × gender	0.00 (0.09)	0.99	0.69–1.43	0.973	0.08 (0.10)	1.11	0.86–1.44	0.435
Social media × age	− 1.66 (0.75)	0.81	0.67–0.97	0.027	− 0.29 (0.69)	0.97	0.85–1.11	0.675
Bedtime (23.00 or later)	0.09 (0.04)	2.81	1.34–5.91	0.012	0.06 (0.05)	1.57	0.74–3.32	0.237
Gender*	0.17 (0.09)	1.88	0.94–3.73	0.067	0.04 (0.09)	1.17	0.61–2.24	0.632
BMI	0.00 (0.07)	1.00	0.92–1.08	0.978	− 0.09 (0.08)	0.95	0.87–1.04	0.248
Age	0.23 (0.07)	1.44	1.15–1.80	0.001	0.12 (0.09)	1.21	0.93–1.56	0.149
Injury	0.03 (0.04)	1.37	0.56–3.34	0.493	0.12 (0.05)	2.90	1.16–7.23	0.020

*0 = boy, 1 = girl. Models are adjusted by bedtime (23.00 or later), BMI (body mass index) and spinal injuries; b = standardized regression coefficient; se = standard error.

Significant values are in bold.

The interaction of screen time and age with NSP incidence between T0–T1 was significant (*b* = -1.85, *se* = 0.83, *p* = 0.027). However, no association was observed between screen time and NSP incidence between T1–T2 (Table 3). Of the different types of screen time, passive gaming time (*b* = 2.75, *se* = 1.31, *p* = 0.036) and social media time (*b* = 1.71, *se* = 0.75, *p* = 0.023) were significantly associated with NSP incidence among the children between T0–T1 (Table 3), but not between T1–T2 (Table 3).

## Discussion

The incidence of NSP was 15% among all at the end of the first follow-up year and 18% at the end of the second follow-up year. Accelerometer-measured MVPA and ST were not associated with the weekly incidence of NSP, but self-reported screen time, especially time spent in social media and passive gaming, were associated with the incidence of NSP in children at the end of the first follow-up year.

This study is among the first to investigate the prospective associations of the incidence of NSP with accelerometer-measured MVPA and ST in school-aged children. Although the previous cross-sectional studies might lead one to expect that PA would associate with NSP incidence in children^8,9,28^, no such association was found. In support, two previous accelerometer-based studies, differed in some respects from ours, also found no association between PA and the incidence or prevalence of NSP. In the study by Wedderkopp et al. (2009) of 9-year-olds (n = 256), the odds for having neck pain three years later did not increase significantly at any PA level, although higher PA was preventive for lower and mid back pain^29^. Participants who developed spinal pain three years later were compared on their baseline PA. Thus, no PA intervention was conducted. In addition, the follow-up was longer than in our study, which means that pain can vary widely without researchers being able to trace it, while the recall period for spinal pain (neck pain, middle back pain, and lower back pain) in a structured interview was shorter at just 1 month^29^. Aartun et al. (2016) also found no cross-sectional or longitudinal associations between accelerometer-measured PA and spinal pain (neck pain, mid back pain, low back pain) in their follow-up data on 11- to 15-year-old adolescents^12^. The biggest difference between their study and ours was that, owing to pain overlap, they collapsed the three spinal areas into one. Therefore, an association between PA and NSP alone was not available for comparison with our results. Moreover, they did not attempt to elucidate the causal relationship, but only the relationship between spinal pain and PA^12^. Franz et al. (2017) reported the intensity of the PA to have an association to spinal pain overall in children 6–12 years of age (n = 1205) in their longitudinal study^30^. The shift from sedentary activity to vigorous PA was associated with increased occurrences of spinal pain and the shift from sedentary to moderate intensity activity appeared to protect against spinal pain^30^. However, the NSP was not separately reported but the pains of the spinal area. Another difference compared to our study was, that the children did not report their pain themselves, but the parents of the children provided the information.

The analysis of self-reported screen time showed an association of overall screen time and time spent in passive gaming and in social media with the incidence of NSP in children during the first follow-up year. For example, one additional hour of screen time per day was associated with a 3.79 -fold increase in the incidence of NSP. Screen time, often thought of as sedentary, can be active, as in the case of exergaming or active mobile games, which were excluded from the analyzed questions describing sedentary behavior and screen time in our study. Clearly, the use of portable smart devices does not always require gaming to meet the active time criterion. Our finding that self-reported screen time was associated with NSP during the first but not the second follow-up year might be partly explained by the development of the children. For example, the use of mobile phones by children and adolescents has been reported to be predominantly a social recreation (73%), and children under the age of 15 appear to have more difficult giving up media devices than older children^31^. This may also be due to the increased awareness of older children about their negative relationship with the media device^31^. Ståhl et al. (2008) have also found fluctuation in neck pain in children^6^.

A recent study found that the use of mobile touch screen device increased the odds for NSP in 10- to 19-year-olds at follow-up one year later, while no association was found between the duration of screen time and NSP^32^. In a Chinese study, also on older adolescents, the use of tablets significantly increased the incidence of NSP^33^. These results suggest that it is important to consider what devices are being used when seeking to understand the association of device use with musculoskeletal symptoms^32^. Bending the upper body over cell phones and other portable devices can lead to increased stress on cervical spine, which in turn can lead to cervical degeneration and other developmental, medical, psychological, and social complications^34^.

A recent Finnish national report (n = 3408) found that problematic 11-, 13- and 15-year-old users of social media (9.4%) suffered twice as much from NSP and headaches than non-problematic users^18^. The problematic social media prevalence was 9.4% and the moderate risk prevalence 33.5%. Problematic use was more prevalent among older users (11.2% in 13- and 15-year-olds, 5.9% in 11-year-olds) and parental monitoring was significantly associated with a lower prevalence of moderate risk and problematic social media use^18^.

The major strengths of the current study were the prospective setting, large sample size, accelerometer-measured PA and ST, and utilization of a well repeatable web-based questionnaire which yields broader information on the context of our study population’s sedentary behavior^35^. Accelerometer-measurements are considered more reliable than self-reports as a measure of activity levels among children^33^. Estimating PA solely with a questionnaire can lead to overestimation of the higher levels of PA and to recall errors^29^. Likewise, it is possible that accelerometers underestimate PA because they fail to reliably detect some forms of PA, such as cycling or strength training, and cannot be used in water-related activities. To eliminate the effects of seasonal variation, the measurements were performed at the same time of year. As a weakness of the study can be mentioned self-reporting for both NSP and screen time variables. The limited use of accelerometers of 7 days based on study design maybe not correspond to a person’s normal physical activity and a typical week in that respect.

As a conclusion, accelerometer-measured PA and ST were not associated with the incidence of NSP in school-aged children at the two-year follow-up. However, self-reported screen time, particularly for the passive gaming and social media use, was associated with NSP incidence at the one-year follow-up. This finding has a novelty value, as the factors that may be important in the recognizing significant NSP problem in school-aged children have now been studied longitudinally. We showed, that screen time at least partially affects children’s NSP symptoms. Time spent in physical activities, in passive gaming and in social media, may compete for children’s use of time, which highlights the role of physical activity in supporting children’s health and well-being. Children with symptoms of NSP may benefit from an assessment of PA and screen time habits.

Finally, in terms of screening time, there is a need to look deeper into the effects, to find information about the different types and contents that can affect the NSP of school-aged children. Research needs to be done specifically to help parents and other adults who are responsible with guiding our school-aged children to the healthy use of technology and understanding the potential disadvantages of that. We may never be able to completely prevent school-aged children’s NSP but we need to understand it, to act consciously to reduce the risk of NSP. Therefore, mechanisms underlying NSP in relation to screen time should be further investigated to develop effective preventive actions for NSP during childhood.

## Supplementary Information

Supplementary Information 1.

Supplementary Information 2.

Supplementary Information 3.

## Data Availability

The data that support the findings of this study are available from [LIKES] but restrictions apply to the availability of these data, which were used under license for the current study, and so are not publicly available. Data are however available from the authors upon reasonable request and with permission of [LIKES].
