# Supplementary material for: Physical activity, screen time and the incidence of neck and shoulder pain in school-aged children
Source: Sci Rep. 2022 Jun 23;12:10635. doi: 10.1038/s41598-022-14612-0 (PMC9226018; doi:10.1038/s41598-022-14612-0)
Supplement: Supplementary file 1 — Supplementary Information 1. [file 41598_2022_14612_MOESM1_ESM.docx]

Supplement 1

SYMPTOMS

How often have you had symptoms in the last three months? (body parts A-I in the picture below)? Mark the appropriate option.

|  | Almost daily | More than once a week | About once a week | About once a month | Seldom or never |
| --- | --- | --- | --- | --- | --- |
| Headache (A) | ⭘ | ⭘ | ⭘ | ⭘ | ⭘ |
| Neck or shoulder pain / ache (B) | ⭘ | ⭘ | ⭘ | ⭘ | ⭘ |
| Upper extremities pain / ache (C) | ⭘ | ⭘ | ⭘ | ⭘ | ⭘ |
| Chest pain / ache (D) | ⭘ | ⭘ | ⭘ | ⭘ | ⭘ |
| Upper back pain / ache (E) | ⭘ | ⭘ | ⭘ | ⭘ | ⭘ |
| Lower back pain / ache (F) | ⭘ | ⭘ | ⭘ | ⭘ | ⭘ |
| Stomach ache (G) | ⭘ | ⭘ | ⭘ | ⭘ | ⭘ |
| Buttocks pain / ache (H) | ⭘ | ⭘ | ⭘ | ⭘ | ⭘ |
| Lower extremities pain / ache (I) | ⭘ | ⭘ | ⭘ | ⭘ | ⭘ |
| Difficulty falling asleep | ⭘ | ⭘ | ⭘ | ⭘ | ⭘ |
| Waking up at night | ⭘ | ⭘ | ⭘ | ⭘ | ⭘ |

Have you injured any of the above-mentioned and pictured pain areas during the previous three months (for example, fallen, stumbled, breached during sport, etc.)

- No
- Yes


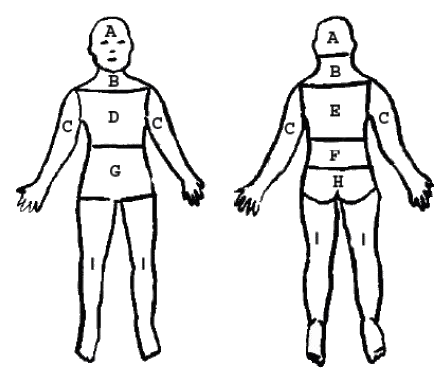
If you answered ”Yes” to the previous question, please indicate the injured body areas. You can choose several options.

- B
- C
- D
- E
- F
- G
- H
- I
