# Supplementary material for: Physical activity, screen time and the incidence of neck and shoulder pain in school-aged children
Source: Sci Rep. 2022 Jun 23;12:10635. doi: 10.1038/s41598-022-14612-0 (PMC9226018; doi:10.1038/s41598-022-14612-0)
Supplement: Supplementary file 2 — Supplementary Information 2. [file 41598_2022_14612_MOESM2_ESM.docx]

Supplement 2

Participants with or without complete baseline data did not differ in the prevalence of NSP (*p*=0.271) or level of ST (*p*=0.227). The participants with incomplete data were more often boys (58.8% vs. 43.9%, *p*<0.001), were older (mean 12.9 years vs. 12.4 years, *p*<0.001), had a higher BMI (mean 19.3 vs. 18.7, *p*=0.020), went to bed later than 23.00 (16% vs. 4%, *p*<0.001) and had a higher level of MVPA (mean 60.5 min/day vs. 52.3 min/day, *p*=0.025) compared to participants with complete data.
