# Supplementary material for: Physical activity, screen time and the incidence of neck and shoulder pain in school-aged children
Source: Sci Rep. 2022 Jun 23;12:10635. doi: 10.1038/s41598-022-14612-0 (PMC9226018; doi:10.1038/s41598-022-14612-0)
Supplement: Supplementary file 3 [file 41598_2022_14612_MOESM3_ESM.odt]

Supplement 3

Interaction between screen time (hours/day) and probability of neck and shoulder pain (NSP) incidence (at least once a week). An association of neck and shoulder pain incidence with screen time (p=0.020) is present between T0–T1 but not between T1–T2 (p=0.272). Note. The predicted probabilities were calculated based on the parameters of the logistic regression model. NSP “rare or never” was the reference category. The model was controlled for age, body mass index, bedtime and injuries in the spinal area.

Interaction between passive gaming time (hours/day) and probability of neck and shoulder pain (NSP) incidence (at least once a week). An association of neck and shoulder pain incidence with passive gaming time (p=0.036) is present between T0–T1 but not between T1–T2 (p=0.226). Note. The predicted probabilities were calculated based on the parameters of the logistic regression model. NSP “rare or never” was the reference category. The model was controlled for age, body mass index, bedtime and injuries in the spinal area. 

Interaction between social media time (hours/day) and probability of neck and shoulder pain (NSP) incidence (at least once a week). An association of neck and shoulder pain incidence with social media time (p=0.023) is present between T0–T1 but not between T1–T2 (p=0.630). Note. The predicted probabilities were calculated based on the parameters of the logistic regression model. NSP “rare or never” was the reference category. The model was controlled for age, body mass index, bedtime and injuries in the spinal area.
